# Food Retention at Endoscopy Among Adults Using Glucagon-Like Peptide-1 Receptor Agonists

**DOI:** 10.1001/jamanetworkopen.2024.36783

**Published:** 2024-10-01

**Authors:** Jason Nasser, Ava Hosseini, Gillian Barlow, Roma Gianchandani, Ali Rezaie, Mark Pimentel, Ruchi Mathur

**Affiliations:** 1 Karsh Division of Gastroenterology and Hepatology, Department of Medicine, Cedars-Sinai Medical Center, Los Angeles, California; 2 Medically Associated Science and Technology Program, Cedars-Sinai Medical Center, Los Angeles, California; 3 Division of Endocrinology, Diabetes, and Metabolism, Department of Medicine, Cedars-Sinai Medical Center, Los Angeles, California

## Abstract

This cross-sectional study investigates the association between glucagon-like peptide-1 receptor agonists and food retention during esophagogastroduodenoscopy.

## Introduction

Glucagon-like peptide-1 receptor agonists (GLP-1RAs) are widely used for diabetes and weight management but are associated with risks for delayed gastric emptying and constipation.^1^ In June 2023, the American Society of Anesthesiologists raised concerns about the potential association between GLP-1RA use and risk of periprocedural aspiration.^2^ We investigated potential associations between GLP-1RAs use and risks of gastric food retention and periprocedural aspiration during esophagogastroduodenoscopies (EGDs) when EGD was combined with colonoscopy and in colonoscopy alone. We additionally evaluated risks of inadequate bowel preparation during colonoscopies.

## Methods

We conducted a retrospective, single-center cross-sectional study of adults undergoing endoscopy between January 1, and June 28, 2023. The Cedars-Sinai Institutional Review Board approved the study, which followed the STROBE reporting guideline, and provided a waiver of informed consent. Individuals taking GLP-1RAs at the time of endoscopy were identified and matched 1:2 with individuals in a control group based on age, body mass index (BMI) subgroup, sex, and procedure. Exclusion criteria included use of prokinetics, surgically modified gastrointestinal anatomy, and exposure to GLP-1RAs within 90 days without actively taking them within 7 days of the procedure. Inadequate preparation was defined as aborting the procedure owing to stool burden or a Boston Bowel Preparation Scale score of less than 6 (total possible score, 9) or 0 (total possible score, 3) in any single bowel segment. Retained solid gastric content was identified based on endoscopist report (eMethods in Supplement 1).

## Results

Among 70 individuals taking GLP-1RAs (mean [SD] age, 62.7 [12.2] years; 36 female) and 139 individuals in the control group (mean [SD] age, 62.7 [12.2] years; 36 female), the mean (SD) BMI was 34.4 (7.2) and 34.4 (7.2), respectively (Table). Among all individuals, 33% underwent EGD, 33% underwent colonoscopy, and 34% underwent both. In the GLP-1RA group, 46% received semaglutide, 30% received dulaglutide, 20% received tirzepatide, and 4% received liraglutide. Food retention occurred in 4 of 23 individuals (17.4%) in the GLP-1RA group undergoing EGD alone (1 moderate, 3 large) vs 0 of 46 individuals in the control group (odds ratio [OR], 21.5; 95% CI, 1.1-414.9; *P* = .01). No food retention was observed in combined EGD-colonoscopies (Figure).

**Table.** Characteristics and Outcomes of 209 Patients

Variable	GLP-1RA group (n = 70)		Control group (n = 139)		*P* value
	Total with data, No.	No. (%)	Total with data, No	No. (%)	
**Characteristic**					
Age, mean (SD), y	70	62.7 (12.2)	139	62.9 (12.3)	NR
EGD only	23	61.5 (14.6)	46	61.8 (14.8)	NR
Colonoscopy only	23	61.6 (10.4)	45	60.5 (10.2)	NR
Combination	24	64.8 (11.5)	48	66.0 (10.9)	NR
BMI, mean (SD)	70	34.4 (7.2)	139	33.2 (5.9)	NR
EGD only	23	32.3 (4.8)	46	31.4 (5.2)	NR
Colonoscopy only	23	34.8 (7.3)	45	33.6 (5.8)	NR
Combination	24	36.0 (8.8)	48	34.6 (6.2)	NR
Sex					
Female	70	36 (51.4)	139	72 (51.8)	NR
EGD only	23	13 (56.5)	46	26 (56.5)	NR
Colonoscopy only	23	8 (34.8)	45	16 (35.6)	NR
Combination	24	15 (62.5)	24	15 (62.5)	NR
Male	70	34 (48.6)	139	67 (48.2)	NR
EGD only	23	10 (43.5)	46	20 (43.5)	NR
Colonoscopy only	23	15 (65.2)	45	29 (64.4)	NR
Combination	24	9 (37.5)	24	9 (37.5)	NR
Race					
African American or Black	70	10 (14.3)	139	25 (18)	.56
American Indian or Alaskan Native	70	2 (2.9)	139	2 (1.4)	.60
Asian	70	1 (1.4)	139	6 (4.3)	.43
Hawaiian or Pacific Islander	70	0 (0)	139	1 (0.7)	>.99
White	70	45 (64.3)	139	93 (66.9)	.76
Other^a^ or not reported	70	12 (17.1)	139	12 (8.6)	.11
Ethnicity					
Hispanic	70	18 (25.7)	139	30 (21.6)	.07
Non-Hispanic	70	50 (71.4)	139	106 (76.3)	.45
Not reported	70	2 (2.9)	139	3 (2.2)	>.99
Obesity (BMI ≥30)	70	50 (71.4)	139	99 (71.2)	.98
EGD only	23	16 (69.6)	46	32 (69.6)	>.99
Colonoscopy only	23	16 (69.6)	45	31 (68.9)	.95
Combination	24	18 (75)	48	36 (75)	>.99
Diabetes	70	58 (82.9)	139	42 (30.2)	<.001
EGD only	23	19 (82.6)	46	9 (19.6)	<.001
Colonoscopy only	23	16 (69.6)	45	18 (40)	.02
Combination	24	23 (95.8)	48	15 (31.3)	<.001
Gastroparesis	70	0	139	1 (0.7)	>.99
EGD only	23	0	46	0	>.99
Colonoscopy only	23	0	45	1 (2.2)	>.99
Combination	24	0	48	0	>.99
Bowel preparation (used 4 L polyethylene glycol)^b^	38	34 (89.5)	73	59 (80.9)	.25
Colonoscopy only	19	17 (89.5)	37	34 (91.9)	.66
Combination	19	17 (89.5)	36	25 (69.4)	.11
**Outcomes**					
Gastric food retention	47	4 (8.5)	94	0	.01
EGD only	23	4 (17.4)	46	0	.006
Combination	24	0	48	0	>.99
Stool retention	47	10 (21.3)	93	6 (6.5)	.009
Colonoscopy only	23	2 (8.7)	45	3 (6.7)	.76
Combination	24	8 (33.3)	48	3 (6.3)	.003
Aspiration	70	0	139	0	>.99
EGD only	23	0	46	0	>.99
Colonoscopy only	23	0	45	0	>.99
Combination	24	0	48	0	>.99

Abbreviations: BMI, body mass index (calculated as weight in kilograms divided by height in meters squared); EGD, esophagogastroduodenoscopy; GLP-1RA, glucagon-like peptide-1 receptor agonist; NR, not reported (parameters on which groups were matched).

^a^ “Other” is an option patients can choose in the electronic medical record system from which data were obtained. No further data on the composition of this group are available.

^b^ For the GLP-1RA group, 47 patients had a colonoscopy, but data were available for only 38 individuals. For the control group, 93 patients had a colonoscopy, but data were available for 73 individuals.

**Figure.**
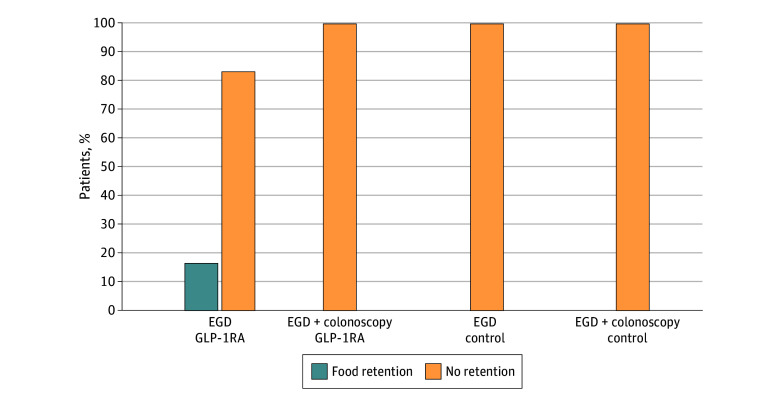
Risk of Food Retention EGD indicates esophagogastroduodenoscopy; GLP-1RA, glucagon-like peptide-1 receptor agonist.

For individuals undergoing colonoscopy or combined EGD-colonoscopies, inadequate bowel preparation was more common in the GLP-1RA than control group (10 of 47 individuals [21.3%] vs 6 of 93 individuals [6.5%]; OR, 3.9; 95% CI, 1.3-11.6). No aspiration events, respiratory distress, or aspiration pneumonia visits occurred.

## Discussion

This cross-sectional study found an association between use of GLP-1RAs and risks of retained gastric contents and inadequate bowel preparation during single endoscopic procedures. While others have explored this topic, this study is unique in examining these risks in EGD alone and when performed with colonoscopy.^3^ Importantly, we observed a low risk of food retention when EGD was combined with colonoscopy. This was suggested in a previous study,^4^ and our results confirm its consistency and reproducibility, supporting a potential utility in preprocedural risk stratification for patients receiving GLP-1RAs for chronic conditions. The protective association of concomitant colonoscopy is likely attributable to the 24-hour clear liquid diet and bowel preparation protocols typically required for colonoscopies.

The association between GLP-1RAs and risk of incomplete bowel preparation is not well described in the literature. Contrary to previous reports, our study identified a clear association between GLP-1RA use and unsatisfactory bowel preparation, which carries significant risks for missed lesions, patient dissatisfaction, and procedure cancellation, with wasted resources.^5,6^

Our results support the value of individualizing recommendations and inform the risk-benefit discussion for preprocedural counseling and same-day counseling when scheduled procedures may be canceled because GLP-1RAs are not held. Importantly, our findings suggest that patients who adhere to colonoscopy preparation guidance may be at a low risk of retained gastric contents and aspiration. However, future studies are necessary to confirm and validate the role of this finding in preoperative risk assessment. Study limitations include a retrospective nature; an inability to match for diabetes or account for other factors, such as glycemia, or examine differential associations of individual GLP-1RAs and doses; and a sample size that precluded analysis of rare events, such as aspiration.

## Notes

Supplement 1.
eMethods.

Supplement 2.
Data Sharing Statement
