# Supplementary material for: Food Retention at Endoscopy Among Adults Using Glucagon-Like Peptide-1 Receptor Agonists
Source: JAMA Netw Open. 2024 Oct 1;7(10):e2436783. doi: 10.1001/jamanetworkopen.2024.36783 (PMC11445686; doi:10.1001/jamanetworkopen.2024.36783)
Supplement: Supplement 1. — eMethods. [file jamanetwopen-e2436783-s001.pdf]

Supplemental Online Content

Nasser J, Hosseini A, Barlow G, et al. Food retention at endoscopy among adults using glucagon-like peptide-1 receptor agonists. *JAMA Netw Open*. 2024;7(10):e2436783.
doi:10.1001/jamanetworkopen.2024.36783

eMethods.

This supplemental material has been provided by the authors to give readers additional information about their work.

© 2024 Nasser J et al. *JAMA Network Open.*

eMethods

GLP-1RAs included semaglutide, tirzepatide, liraglutide, dulaglutide, exenatide, and lixisenatide. Matching by age was ± 5 years. Matching by BMI subgroups entailed the following grouping: <18.5, 18.5-24.9, 25.0-29.9, 30.0-34.9, and ≥35.0 kg/m^2. During the study period, the institutional protocol included an 8-hour preprocedural fast and the colonoscopies further required a 3-day low-residue and 1-day clear-liquid diet, while there was no protocol for preprocedural management of GLP-1RAs. Statistical analyses were performed using IBM SPSS Statistics v27.0 (IBM Corp., Armonk, NY, USA). Study staff reviewed electronic medical records (EMR) to obtain demographic information, including race and ethnicity. All demographic information was self-reported by participating individuals. The race and ethnicity options listed are as presented in the institutional EMR system, Epic. “Other” is a listed option that participants can choose. Chi-square or Fisher's exact tests were employed to compare categorical variables between groups, and odds ratios (OR) with 95% confidence intervals (95% CI) were calculated to assess the strength and precision of associations. For analyses involving zero cell counts, the Haldane-Anscombe correction was applied to calculate adjusted OR and 95% CI, ensuring appropriate handling of small sample sizes and zero counts. For continuous variables, independent samples t-tests were used to compare means between groups. All statistical tests were two-tailed, and significance was set at $p < 0.05$. The study report was prepared following the STROBE reporting guidelines (https://www.equator-network.org/reporting-guidelines/strobe/).

© 2024 Nasser J et al. *JAMA Network Open.*
