# Supplementary material for: Food Retention at Endoscopy Among Adults Using Glucagon-Like Peptide-1 Receptor Agonists
Source: JAMA Netw Open. 2024 Oct 1;7(10):e2436783. doi: 10.1001/jamanetworkopen.2024.36783 (PMC11445686; doi:10.1001/jamanetworkopen.2024.36783)
Supplement: Supplement 2. — Data Sharing Statement [file jamanetwopen-e2436783-s002.pdf]

Data Sharing Statement

Nasser. Food Retention at Endoscopy Among Adults Using Glucagon-Like Peptide-1 Receptor Agonists. *JAMA Netw Open*. Published online October 1, 2024. doi:10.1001/jamanetworkopen.2024.36783

Data
Data available: No
